# Editorial: Plant Glycobiology - A Sweet World of Glycans, Glycoproteins, Glycolipids, and Carbohydrate-Binding Proteins

**DOI:** 10.3389/fpls.2021.751923

**Published:** 2021-09-03

**Authors:** Georg J. Seifert, Richard Strasser, Els J. M. Van Damme

**Affiliations:** ^1^Department of Applied Genetics and Cell Biology, University of Natural Resources and Life Sciences, Vienna, Austria; ^2^Department of Biotechnology, Ghent University, Ghent, Belgium

**Keywords:** protein glycosylation, post-translational modification, lectin, carbohydrate, cell wall polysaccharide

## Introduction

Plants synthesize a wide variety of glycan structures which play essential roles during plant development and contribute to many diverse processes. These glycans function as structural components in the plant cell wall, assist in the folding and secretory trafficking of nascent proteins, act as signaling molecules in stress and plant defense responses, or serve within the energy metabolism of a plant (Strasser, 2016). Studying the biological roles of plant glycans enables a better understanding of plant growth and development under various environmental conditions in order to protect plants and exploit them for agriculture, forestry, or biotechnological products (De Coninck et al., 2021).

This Research Topic consists of 32 papers, including 10 review papers, 21 original research articles and 1 perspective article, and provides an overview of the latest research, methodologies and applications related to the fascinating world of plant glycobiology.

## *N-*Glycans and Their Role in Plant Development

*N-*glycosylation is an abundant posttranslational modification of proteins entering the secretory pathway in all eukaryotes. It is well-established that the attached *N-*glycans have an impact on protein folding and quality control processes. The function of specific *N*-linked glycan modifications is, however, still obscure in plants (Nagashima et al., 2018). In this Research Topic several original research papers and reviews report new findings and highlight important aspects of specific *N*-glycan modifications.

The review by Zhang et al. highlights the role of a distinct oligomannosidic *N*-glycan as a signal for termination of quality control processes and initiation of ER-associated degradation (ERAD). The authors provide detailed insights into conserved and unique features of mammalian, yeast and plant α-mannosidases involved in ERAD of misfolded glycoproteins. While a coherent picture is emerging for the early steps of the ERAD pathway, later steps that involve retrotranslocation of the misfolded glycoprotein to the cytosol and clearance are still poorly characterized. Prior to degradation by the proteasome, ERAD substrates are predicted to undergo deglycosylation by the sequential action of two cytosolic enzymes, peptide:*N-*glycanase and endo-β-*N*-acetylglucosaminidase, resulting in the release of oligomannosidic free *N*-glycans from the misfolded glycoproteins. Maeda et al. have purified rice endo-β-*N-*acetylglucosaminidase and show that the substrate specificity of the native and recombinant enzyme are almost the same. The same group reports that oligomannosidic free *N-*glycans are present in microsomes isolated from pumpkin hypocotyls which leads to the interesting model that oligomannosidic free *N*-glycans could be taken up by the ER, from where they are secreted through the Golgi to the extracellular space (Katsube et al.). Like glycoproteins, the free *N*-glycans are processed in the Golgi, but the biological relevance of secreted complex-type free *N*-glycans is unknown.

Tobacco BY-2 cell suspensions are an established model system for plant cell biology (Nagata et al., 1992) and used as production platform for recombinant proteins (Santos et al., 2016). Herman et al. use CRISPR/Cas9 genome editing to produce homogenous mannosidic *N*-glycans on BY-2 cell produced glycoproteins. Intriguingly, the loss-of-function approach reveals a new aspect of the *N*-glycan processing pathway. Knockout of *N*-acetylglucosaminyl transferase I (GNTI), the key enzyme for complex *N*-glycan formation, is not sufficient to eliminate all *N*-glycans with core fucose suggesting that core fucosylation is not strictly dependent on the GlcNAc residue transferred by GNTI. In tomato, GNTI and complex *N*-glycans are crucial for development. Knockdown of GNTI in tomato causes necrotic fruit-attached stalks, early fruit drop, and patchy fruit ripening (Kaulfürst-Soboll et al.). By contrast, only subtle phenotypes are observed when hybrid *N*-glycans are generated by silencing of Golgi α-mannosidase II, which catalyzes the subsequent processing reaction in the pathway.

Using a label-free quantitative proteomics approach Liu, Niu et al. identify proteins that are differentially regulated under salt stress in *Arabidopsis thaliana* mutants lacking complex N-glycans and uncover several glycoproteins that could be involved in salt stress tolerance. Frank et al. examine the effect of synthetic phytohormones on root hair formation in Arabidopsis mutants with blocked or altered complex *N*-glycan processing. The lack of complex *N*-glycans in the *gntI* mutant causes increased root hair elongation and hypersensitivity to a synthetic auxin when compared to wild-type roots. Lewis A containing structures are the most elaborate complex-type *N*-glycans found in plants (Strasser et al.). Strikingly, Arabidopsis mutants with impaired biosynthesis of Lewis A structures display also elongated root hairs demonstrating for the first time a biological role of Lewis A containing *N-*glycans (Frank et al.). In Arabidopsis, the occurrence of Lewis A containing glycoproteins is tissue-specific with high amounts in stems and siliques and low levels in roots (Strasser et al., 2007). Beihammer, Maresch, Altmann, Van Damme et al. use the Lewis A specific JIM84 antibody to identify by affinity purification and nano-LC-MS analysis Lewis A containing glycoproteins from Arabidopsis, rice and *Nicotiana benthamiana* and show that they may be associated with different functions in different plant species. By this approach they also demonstrate that the Lewis A structures are highly conserved in different Arabidopsis accessions. Using a similar approach, the identification of Lewis A-containing glycoproteins from root hairs may in the future pave the way to reveal the mechanisms underlying the altered auxin sensitivity and the role of complex *N*-glycans in the regulation of root hair elongation.

While the formation of Lewis A type structures takes place in the trans-Golgi, truncated or paucimannosidic *N*-glycans are generated in a downstream compartment or in the apoplast by removal of one or two terminal GlcNAc residues. Alvisi et al. report that the three *N. benthamiana* β-hexosaminidases (HEXO1–HEXO3) have distinct substrate specificities. HEXO1 and HEXO3 cleave off GlcNAc residues from complex *N*-glycans and both, HEXO2 and HEXO3, are capable of GalNAc trimming from *N*-glycans. Plant *N*-glycans are normally not decorated with GalNAc residues, but the study has important implications for glycoengineering approaches aiming at the generation of homogenous *N-* and *O-*linked glycan structures found in helminths or mammals (Castilho et al., 2012; Wilbers et al., 2017).

Compared to higher plants, little is still known about the biosynthesis of *N-* and *O-*glycans in microalgae which display a remarkable structural diversity. Recent advances in characterization of *N*-glycans isolated from different microalgae species are nicely summarized in the review by Mathieu-Rivet et al. Differences in *N-*glycan precursor biosynthesis and processing steps in the Golgi result in an unexpected variety of *N-*glycan structures that appear characteristic for microalgae. Mócsai, Kaehlig et al. add another facet to the complexity of major *N*-glycans found in different microalgae. Using a combination of independent techniques including MALDI-TOF-MS, linkage analysis by GC-MS and NMR, the authors reveal the structural differences of uncommon isobaric *N*-glycan structures that are derived from phylogenetically divergent microalgae species.

## *O-*Glycosylation: Arabinogalactan Proteins, Hydroxyproline-Rich Glycoproteins and Cell Wall Polymers

Another notable aspect of this Research Topic is the interest in protein *O-*glycosylation and hydroxyproline-rich glycoproteins (HRGPs) in the cell wall. An impressive six articles review protein glycosylation in plants, summarizing our present knowledge of both *N-* and *O-*glycosylation in higher plants (Strasser et al.) and microalgae (Mathieu-Rivet et al.), as well as highlighting structural-functional aspects of arabinogalactan-proteins (AGPs) (Seifert, Silva et al.) and of extensins, with emphasis on arabinosylation (Petersen et al.) and oxidative crosslinking (Mishler-Elmore et al.), respectively.

Most *O-*glycans in plants, including microalgae (Mathieu-Rivet et al.) are attached to hydroxyproline (Hyp), an amino acid that is generated post-translationally by the action of prolyl-4-hydroxylases (P4H), as reported in two research articles in this Research Topic. Mócsai, Göritzer et al. take a long overdue new attempt at the substrate specificity of P4H isoforms, albeit with the result that four individual *N. benthamiana* P4H isoforms do not show differences in substrate selectivity in a non-plant expression system. In contrast, the study by Konkina et al. demonstrates a specific role for the *A. thaliana P4H3* gene in AGP abundance and tolerance to hypoxia. Hence, the mechanism of P4H selectivity remains an open question.

The build-up of Hyp-linked *O*-glycan structures requires a multitude of glycosyl transferases several of which are presently known (Strasser et al.; Petersen et al.; Silva et al.). In this issue, the *A. thaliana GALT8* locus is described to encode an enzyme with β-(1,3) galactosyl transferase activity *in vitro* (Narciso et al.). This activity is needed for the elaboration of arabinogalactan (AG) type II structures found on all AGPs and some pectic polymers. Thus, GALT8's biochemical function is consistent with the global growth phenotype of *galt8* single mutants. However, the weakness of the *galt8* phenotype might be due to partial redundancy with the *KNS4* and the *At1g77810* β-(1,3) galactosyl transferase encoding loci. Type II AG on some AGPs contains α-(1,2) linked terminal fucose and it is believed that the Arabidopsis *FUT4* and *FUT6* loci encode the corresponding fucosyl transferases (Strasser et al.; Silva et al.). New biochemical *in vitro* work presented in this Research Topic in principle widens the activities of FUT4 and FUT6 to any α-(1,3) and α-(1,5) linked terminal arabinofuranose which includes type II AG but also non-AGP like oligosaccharides (Soto et al.).

One subfamily of AGP is defined by their fasciclin1 domains that have been hypothesized to interact with proteins or carbohydrates. These FASCICLIN LIKE AGPs (FLAs) are known to act in different developmental roles and two research articles demonstrate non-redundant roles for two *A. thaliana FLA* loci encoding group B glycoproteins that in contrast to most FLAs do not contain GPI-anchors. The study by Liu, MacMillan et al. shows that *FLA16* expression correlates with secondary cell wall formation and that the FLA16 protein is a moderately glycosylated protein localized to the plasma membrane and the apoplast. Intriguingly, the *fla16* loss of function mutation lacks normal stem strength and cellulose content indicating a role of *FLA16* in secondary cellulose formation similar to what has previously been described for *FLA11* and *-12* (MacMillan et al., 2010). Another group B *FLA* locus called *FLA18* is the subject of the paper by Allelign Ashagre et al.. In contrast to the secondary growth-related *FLA16, FLA18* expression correlates with expansion growth in roots i.e. primary cell wall formation. The root swelling *fla18* phenotype that depends on sucrose-levels in the growth medium is reminiscent of the *fla4* mutant but specific to lateral roots. However, the genetic interaction between the loci is synergistic suggesting independent roles of the two FLAs in the process of root elongation. Despite these intriguing first descriptions of single loss of function phenotypes of group B *fla* mutants, the mode of action of the entire FLA family remains largely obscure.

Finally, two research papers present genome-wide surveys of HRGPs (Abedi et al.) and potentially glycan binding, lectin-domain proteins (Petrova et al.) in relation to cell wall formation in the fiber crops poplar and flax, respectively. Both studies conclude that expression of many cell wall proteins is closely correlated with the formation of polysaccharides of different cell wall types, which is also in line with the two aforementioned functional studies on *FLA16* and *FLA18*.

In contrast to the raft of papers on cell wall proteins, relatively few contributions in this Research Topic deal with cell wall polysaccharide biosynthesis. The review by Zabotina et al. surveys the emerging theme of complex formation in the biosynthesis of cell wall matrix polymers in the Golgi. While this field is experimentally challenging it opens up the possibility of regulating polysaccharide abundance and structure at the cellular level and is of equal importance as the cellulose synthase complex formation and motility to fully understand cell wall biosynthesis. However, also the search for new components in cell wall polysaccharide biosynthesis is far from over as exemplified by the knock-out study of the only orthologous gene to pectic rhamnogalacturonan I (RG I) backbone synthesizing rhamnosyl transferase (RRT) in liverwort *Marchantia polymorpha*, published in this Research Topic (Wachananawat et al.). While MpRRT1 should be essential for RG I biosynthesis the *mprrt1* mutant still contains 80% of the normal RG I content pointing to a contribution of other RG I backbone synthesizing loci that remain to be identified.

## Protein-Carbohydrate Interactions, up to Applications

The specific interaction between proteins such as carbohydrate-active enzymes and lectins, and carbohydrate structures is at the forefront plant glycobiology. Consequently, several papers in this Research Topic aim at the (functional) characterization of glycosyl transferases, lectins, GPI anchor modifications and their importance for plant development.

Beihammer, Maresch, Altmann et al. provide insights into the biosynthesis of the lipid-linked glycan backbone that is subsequently transferred to proteins during the attachment of a GPI anchor. MS analysis and glycosidase digestion show that the core glycan of a GPI-anchor derived from a reporter protein carries an additional galactose residue that is likely transferred in the Golgi.

Romero-Pérez et al. report in their study that overexpression of the chimeric lectin F-box Nictaba helps Arabidopsis plants to cope with bacterial infection from *Pseudomonas syringae*. In addition, higher levels of anthocyanins are accumulating in overexpression lines compared to wild-type plants and knock-out lines.

Putative lectins are the subject of a bioinformatic transcriptome analysis of gene expression in various flax tissues producing different types of cell walls. From this analysis it is predicted that the multiple lectin mediated processes take place in plant cell walls throughout plant development (Petrova et al.).

Functional studies are an important step toward possible applications of glycobiology research. Zhang and Showalter give an overview of the principles and use of CRISPR/Cas9 to perform functional analysis of genes important for plant cell wall biosynthesis. While the application of genome editing tools to questions related to cell wall biology is still limited to a few examples, the authors show that because of the high specificity and the possibility for multi-gene targeting CRISPR/Cas9 is an ideal tool to uncover biological functions of genes, which can be extended and applied to many other genes encoding carbohydrate-modifying enzymes, and beyond. Shin et al. used the *N. benthamiana* platform for functional expression of the SARS-CoV-2 receptor binding domain. Though *N*-glycosylation of the receptor binding domain is important for proper folding, the *N*-glycan processing did not affect its functionality.

In the past decades, plants have become a very attractive platform for recombinant protein production of glycoproteins. Consequently, several model plants have been glyco-engineered to enable synthesis of human *N-*glycan structures in plants (Montero-Morales and Steinkellner, 2018; Margolin et al., 2020). Bohlender et al. were successful in establishing stable protein sialylation in *Physcomitrella patens*, creating opportunities for this moss to be used as a plant-based biopharmaceutical production platform. In their review paper Margolin et al. highlight recent progress in glycoengineering and the engineering of glycosylation-directed folding pathways in plants, aiming for use in the production of recombinant viral glycoprotein vaccines (Gitzinger et al., 2009; Castilho et al., 2010).

Although many papers in this Research Topic still focus on *A. thaliana* as a model plant, several other models, such as microalgae, *P. patens, M. polymorpha, N. benthamiana*, and *N. tabacum, Populus*, flax, tomato, and rice are now also being used in plant glycobiology studies. Clearly this Research Topic highlights the progress made in the field of plant glycobiology, and emphasizes the importance of glycosylated molecules and polysaccharides for plant growth and development.

## Author Contributions

All authors contributed equally to this manuscript and approved the final version.

## Conflict of Interest

The authors declare that the research was conducted in the absence of any commercial or financial relationships that could be construed as a potential conflict of interest.

## Publisher's Note

All claims expressed in this article are solely those of the authors and do not necessarily represent those of their affiliated organizations, or those of the publisher, the editors and the reviewers. Any product that may be evaluated in this article, or claim that may be made by its manufacturer, is not guaranteed or endorsed by the publisher.
